# Premature Myocardial Infarction (MI) Complicated by Cardiac Arrest Secondary to Essential Thrombocythaemia (ET)

**DOI:** 10.7759/cureus.94761

**Published:** 2025-10-17

**Authors:** Ahmed Moemen A Omar, Charo Bruce, Nesan Shanmugam, Faisal Khan

**Affiliations:** 1 Cardiology, St George's University Hospitals NHS Foundation Trust, London, GBR

**Keywords:** atherosclerosis, essential thrombocythaemia, myeloproliferative neoplasm (mpn), out-of-hospital cardiac arrest, premature coronary artery disease

## Abstract

This article describes a case of premature myocardial infarction (MI) in a young individual, complicated by cardiac arrest and ultimately attributable to essential thrombocythaemia (ET). The report emphasises the necessity of considering underlying haematological disorders as unconventional risk factors for cardiovascular events, particularly in younger patients without traditional risk factors. Prompt recognition and multidisciplinary management are imperative to optimise clinical outcomes in such atypical presentations.

## Introduction

Premature myocardial infarction (MI) is generally defined as a heart attack occurring in men aged 18-55 years and women aged 18-65 years [1-4]. The incidence of MI in younger populations is rising, with registry data showing a significant increase in very young patients (<40 years) over the past decade. Importantly, both short- and long-term outcomes in this cohort are comparable to those in patients aged 41-50 years [1-4].

The primary risk factors for early MI remain well established, including diabetes mellitus, smoking, dyslipidaemia and obesity. However, additional haematological disorders, particularly essential thrombocythaemia (ET) and polycythaemia vera (PV), also significantly increase the risk of cardiovascular events, including MI [5]. The reported frequency of MI in patients with ET is <10%, and it is rarely the initial clinical manifestation [5].

ET is a chronic myeloproliferative neoplasm (MPN) characterised by the clonal overproduction of platelets, most frequently driven by the JAK2 V617F mutation, which is present in approximately 60% of cases [4,6]. Clinically, ET can present with vasomotor symptoms such as headache, visual disturbance or erythromelalgia, but it may also initially manifest with thrombotic events, including stroke or MI [5].

The JAK2 V617F mutation contributes to the risk of premature MI by potentiating both prothrombotic and proinflammatory pathways. In addition to promoting platelet proliferation, it induces endothelial dysfunction, macrophage activation and erythrophagocytosis, thereby accelerating atherosclerosis and vascular inflammation [6,7].

## Case presentation

**Figure 1:**
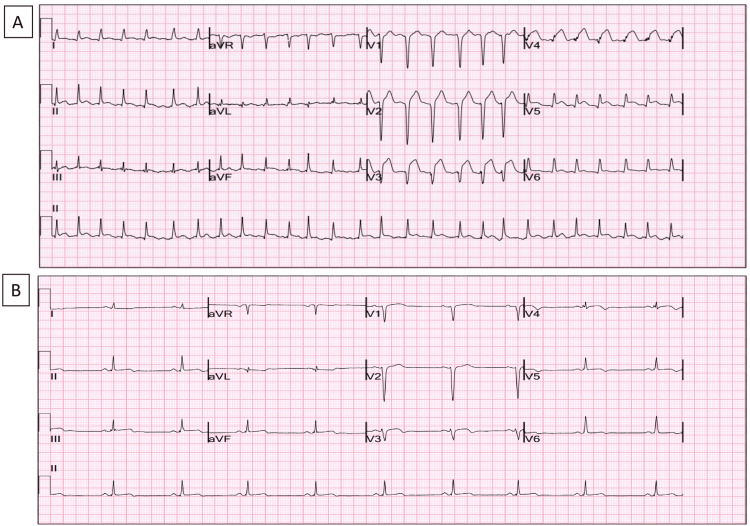
Hospital admission and post PPCI 12-lead ECGs. A 12-lead electrocardiogram (ECG) displayed anterior ST elevation myocardial infarction (MI) on admission to the hospital (A) that resolved later post primary percutaneous coronary intervention (PPCI) (B).

A young man in his early 20s, with no significant medical history, experienced a sudden collapse and cardiac arrest while commuting to work. Prompt bystander cardiopulmonary resuscitation (CPR) was administered successfully, and paramedics delivered five direct current cardioversion (DCCV) shocks to address ventricular fibrillation (VF) and return of spontaneous circulation (ROSC). His initial electrocardiogram (ECG) revealed a significant anterior ST elevation myocardial infarction (STEMI) (Figure 1A), prompting his transfer to our cardiac tertiary centre for primary percutaneous coronary intervention (PPCI). Upon arrival at the hospital, he was haemodynamically stable without respiratory or cardiovascular support; his Glasgow Coma Scale was 15/15.

**Figure 2:**
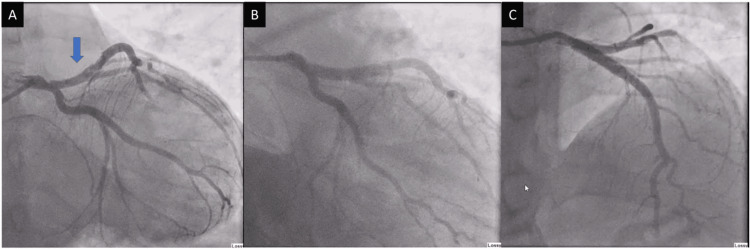
Emergent coronary angiogram. Emergent coronary angiogram demonstrated thrombotic plaque rupture at the ostium of the left anterior descending artery (LAD) with distal vessel occlusion (A) that was treated with IVUS-guided, deployed drug-eluting stent (DES) (B and C). IVUS: intravascular ultrasound

An emergency coronary angiogram demonstrated thrombotic plaque rupture at the ostium, with distal vessel occlusion involving the origin of the left anterior descending artery (LAD) (Figure 2A). Recanalisation could not be achieved despite thrombus aspiration and the administration of an intravenous bolus of tirofiban, a glycoprotein IIb/IIIa inhibitor. The patient subsequently underwent intravascular ultrasound (IVUS)-guided primary percutaneous coronary intervention (PPCI), during which a drug-eluting stent (DES) was successfully deployed across the affected segment. No additional lesions were identified (Figure 2B, 2C).

Initial blood tests (Table 1) revealed markedly elevated white blood cell (WBC) and platelet counts. Although these parameters gradually improved during hospitalisation, the platelet count remained persistently elevated. Genetic testing confirmed the presence of the JAK2 V617F mutation, while BCR-ABL, CALR, and MPL mutation analyses were negative.

**Table 1:** Summary of key laboratory investigations. LDH: lactate dehydrogenase

Parameter	Value	Reference Range	Interpretation
White blood cell (WBC) count	35 × 10⁹/L	4.5-11.0 × 10⁹/L	Elevated
Platelet count	1031 × 10⁹/L	150-450 × 10⁹/L	Elevated
Lipoprotein(a)	<7 nmol/L	<7 nmol/L	Normal
Low-density lipoprotein (LDL) cholesterol	1.34 mmol/L	<3.4 mmol/L	Normal
Total cholesterol	2.6 mmol/L	3.3-5.2 mmol/L	Reduced
Homocysteine	<5.5 µmol/L	5-15 µmol/L	Normal
Ferritin	595 µg/L	30-400 µg/L	Elevated (acute-phase response)
Serum iron	9 µmol/L	10-30 µmol/L	Reduced
Transferrin	2.03 g/L	2.0-3.6 g/L	Low-normal
Transferrin saturation	18%	20%-50%	Reduced
LDH	255 U/L	<250 U/L	Mildly elevated
JAK2 V617F mutation	Positive	-	Detected
BCR-ABL mutation	Negative	-	Not detected
CALR mutation	Negative	-	Not detected
MPL mutation	Negative	-	Not detected
Platelets (nine-month review)	316 × 10⁹/L	150-450 × 10⁹/L	Normal
WBC (nine-month review)	4.9 × 10⁹/L	4.5-11.0 × 10⁹/L	Normal

Additional investigations demonstrated normal lipoprotein(a) and low-density lipoprotein (LDL) cholesterol levels, reduced total cholesterol and normal homocysteine concentrations. Iron studies indicated low circulating iron with elevated ferritin and preserved transferrin, consistent with an acute-phase response rather than true iron deficiency. Lactate dehydrogenase (LDH) was mildly elevated.

**Figure 3:**
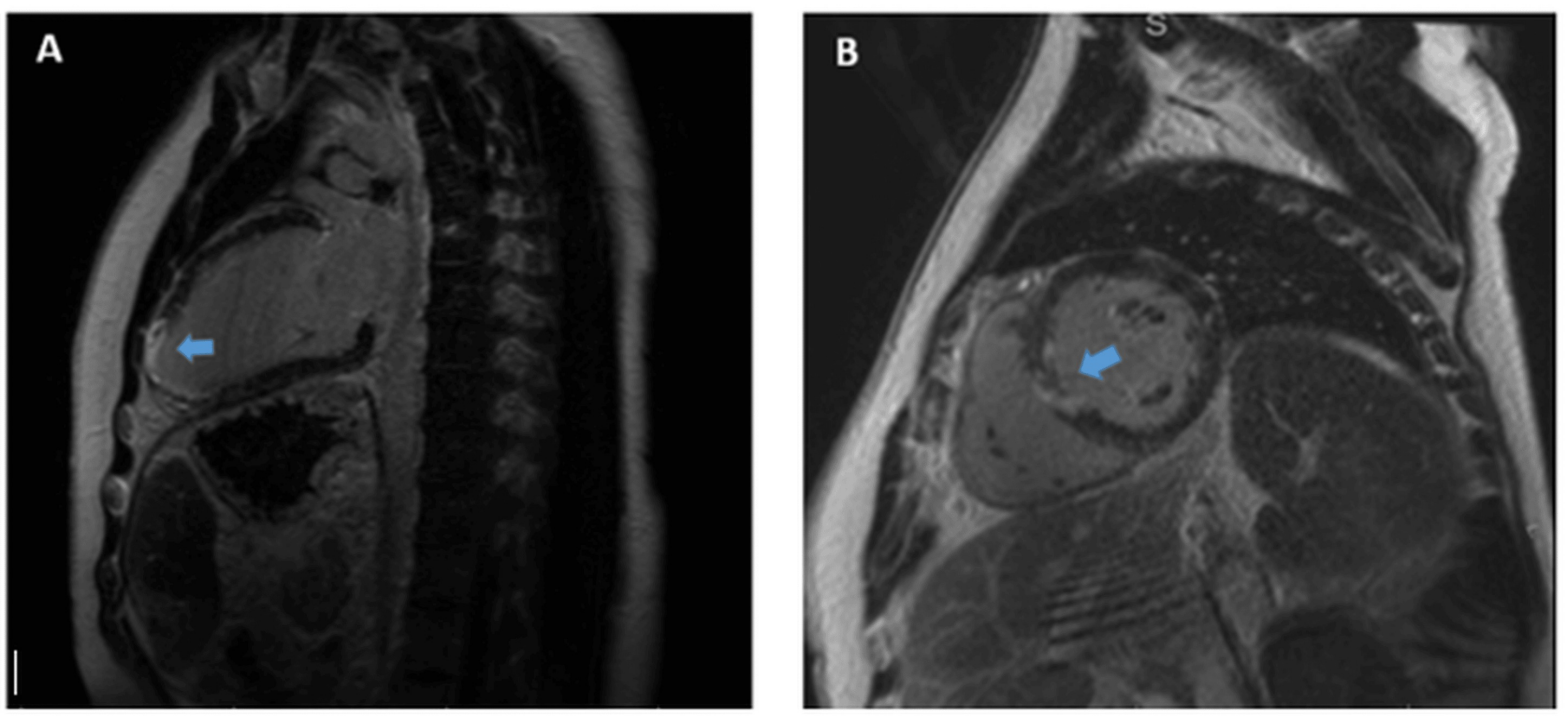
Cardiac magnetic resonance (CMR) with late gadolinium enhancement (LGE). (A) Extensive apical and anterior enhancement indicating ischaemic scar. (B) Subendocardial to transmural anterior and septal enhancement indicating LAD-territory infarction. LAD: left anterior descending artery

Bedside echocardiography at admission demonstrated the severe impairment of left ventricular systolic function with an ejection fraction (EF) of 35%, without significant valvular disease. By discharge, repeat echocardiography revealed improvement, with an EF of 50%. Cardiac magnetic resonance imaging (CMR) confirmed infarction involving the basal-mid inferior septum and all apical segments, with a left ventricular EF of 48% and a normal right ventricular size but mildly impaired systolic function (EF: 50%) (Figure 3A, 3B).

During hospitalisation, the patient developed transient episodes of melena. Upper gastrointestinal endoscopy did not identify a bleeding source, and abdominal ultrasonography demonstrated normal liver and spleen size. As no further episodes of melena were reported, conservative management was adopted, and capsule endoscopy was not performed.

On further enquiry, the patient reported being a non-smoker with no history of recreational drug use and an average alcohol intake of two units per week. Notably, he had experienced intermittent chest pain on the night prior to his collapse. He denied recent fever, weight loss, night sweats or lymphadenopathy. His family history was significant, with his maternal grandfather sustaining a myocardial infarction (MI) in his late 40s and his mother at the age of 54, suggesting a possible genetic predisposition.

The patient was subsequently reviewed by the haematology team, with an initial impression of reactive thrombocytosis secondary to myocardial infarction (MI), supported by gradual improvement in platelet and white blood cell counts during his 13-day admission. However, the platelet count remained elevated. After discharge, genetic testing confirmed a JAK2 V617F mutation, and the case was subsequently discussed at a multidisciplinary team (MDT) meeting, where a consensus diagnosis of JAK2-mutated essential thrombocythaemia (ET) was established.

Differentials considered included reactive thrombocytosis, later excluded following the JAK2 result, and other myeloproliferative neoplasms (MPNs) such as polycythaemia vera (PV) and primary myelofibrosis (PMF). These entities often require cytoreductive therapy (e.g., hydroxycarbamide) in patients with thrombotic events such as MI.

A bone marrow biopsy was recommended to establish a baseline assessment; however, this was deferred until the patient completed 12 months of dual antiplatelet therapy (DAPT) following percutaneous coronary intervention (PCI), owing to the associated bleeding risk.

Following primary percutaneous coronary intervention (PPCI), the patient was commenced on dual antiplatelet therapy (DAPT), consisting of aspirin 75 mg once daily and ticagrelor 90 mg twice daily, together with gastroprotection using lansoprazole 30 mg once daily. In addition, heart failure prognostic medications were initiated at low doses and gradually up-titrated during hospital admission, including bisoprolol and dapagliflozin, with plans to introduce further heart failure therapies in the outpatient setting.

Following PCI, the patient's electrocardiogram (ECG) normalised (Figure 1B), and his recovery progressed steadily. He was discharged home with planned follow-up appointments in both cardiology and haematology.

Four months after the acute event and following the completion of sperm banking arrangements, the patient was commenced on hydroxycarbamide at a daily dose of 500 mg. Pegylated interferon-α was avoided due to the patient's marked needle phobia, while anagrelide was contraindicated in the post-STEMI setting because of its potential cardiotoxic effects.

At the nine-month review, after the initiation of hydroxycarbamide therapy, both platelet and white blood cell counts had returned to normal (platelets, 316 × 10⁹/L; WBC, 4.9 × 10⁹/L). A diagnostic bone marrow biopsy is scheduled once 12 months of dual antiplatelet therapy have been completed, in order to reduce bleeding risk.

## Discussion

Myocardial infarction (MI) remains a leading global cause of morbidity and mortality. While traditionally associated with older age and conventional risk factors, such as diabetes, smoking, dyslipidaemia and obesity, premature MI is increasingly recognised in younger patients [1-4]. In such cases, non-traditional contributors must be considered.

One such contributor is essential thrombocythaemia (ET), a chronic myeloproliferative neoplasm characterised by clonal platelet overproduction and qualitative platelet dysfunction. Although ET is an established risk factor for thrombotic events, its role in premature MI, particularly in the absence of traditional risk factors, is under-recognised [5].

Linking ET and premature MI

ET increases thrombotic risk through mechanisms beyond elevated platelet counts. Platelets in ET demonstrate functional abnormalities that predispose to spontaneous aggregation and intravascular thrombosis [5]. Clinically, MI occurs in <10% of patients with ET and is rarely the first manifestation of the disease [5]. Nevertheless, when MI presents in a young patient without conventional risk factors, ET should be considered as a possible underlying aetiology.

The JAK2 V617F mutation, present in approximately 60% of ET cases, contributes to thrombosis through both proliferative and proinflammatory mechanisms [4,6]. It promotes endothelial dysfunction, macrophage activation and vascular inflammation, thereby accelerating atherogenesis and increasing plaque vulnerability [6,7].

ET as a non-traditional risk factor for atherosclerosis

While hypertension, diabetes and dyslipidaemia remain dominant drivers of atherosclerosis, non-traditional contributors such as ET are increasingly recognised in premature disease. ET-associated systemic inflammation, combined with abnormal platelet activity and cytokine release, fosters endothelial injury and plaque progression [4,6]. Evidence suggests that the JAK2 V617F mutation amplifies oxidative stress and leukocyte activation, thereby accelerating vascular ageing and destabilising plaques [6,7].

Impact on plaque vulnerability and stability

Acute coronary syndromes typically result from the rupture of vulnerable plaques with thin fibrous caps and high inflammatory cell content [8,9]. ET may augment this vulnerability via multiple mechanisms, notably through inflammatory activation. In JAK2-mutated ET, persistent inflammation promotes macrophage-derived metalloproteinase activity, resulting in the degradation of the fibrous cap [6].

Additionally, microthrombosis, caused by elevated and dysfunctional platelets, facilitates intraplaque microthrombi, predisposing to intraplaque haemorrhage and rupture [5,9].

Furthermore, emerging evidence suggests that JAK2 V617F-positive essential thrombocythaemia (ET) contributes to accelerated coronary calcium deposition and increased arterial stiffness, thereby augmenting cardiovascular risk through vascular remodeling processes. Collectively, these mechanisms highlight ET's capacity to destabilise atherosclerotic plaques, transforming them from stable to rupture-prone lesions and consequently elevating the likelihood of acute coronary events [7].

In the presented case, the premature myocardial infarction (MI) appears to have been primarily mediated by ET-associated atherogenesis and plaque destabilisation, driven by chronic inflammation and endothelial dysfunction, rather than by a purely thrombotic mechanism.

The recognition of this association is crucial for timely diagnosis and appropriate management. Further research is required to elucidate the molecular mechanisms by which ET accelerates vascular disease, with the aim of developing targeted therapies to improve cardiovascular outcomes in this distinct patient group.

## Conclusions

Premature myocardial infarction in patients without conventional cardiovascular risk factors should prompt evaluation for alternative aetiologies. Essential thrombocythaemia, particularly in the presence of the JAK2 V617F mutation, represents a significant non-traditional risk factor that contributes not only to thrombosis but also to accelerated atherogenesis and plaque vulnerability through proinflammatory and endothelial-disruptive mechanisms.

## References

[1] De Sutter J, De Bacquer D, Kotseva K, Sans S, Pyorala K, Wood D, De Backer G (2003). Screening of family members of patients with premature coronary heart disease: results from the EUROASPIRE II family survey. Eur Heart J.

[2] Kotseva K, Wood D, De Backer G, De Bacquer D, Pyörälä K, Keil U (2009). EUROASPIRE III: a survey on the lifestyle, risk factors and use of cardioprotective drug therapies in coronary patients from 22 European countries. Eur J Cardiovasc Prev Rehabil.

[3] Lloyd-Jones DM, Nam BH, D'Agostino RB Sr (2004). Parental cardiovascular disease as a risk factor for cardiovascular disease in middle-aged adults: a prospective study of parents and offspring. JAMA.

[4] Tefferi A, Vainchenker W (2011). Myeloproliferative neoplasms: molecular pathophysiology, essential clinical understanding, and treatment strategies. J Clin Oncol.

[5] Rossi C, Randi ML, Zerbinati P, Rinaldi V, Girolami A (1998). Acute coronary disease in essential thrombocythemia and polycythemia vera. J Intern Med.

[6] Wang W, Liu W, Fidler T (2018). Macrophage inflammation, erythrophagocytosis, and accelerated atherosclerosis in Jak2 (V617F) mice. Circ Res.

[7] Anžič Drofenik A, Vrtovec M, Božič Mijovski M, Sever M, Preložnik Zupan I, Kejžar N, Blinc A (2020). Progression of coronary calcium burden and carotid stiffness in patients with essential thrombocythemia associated with JAK2 V617F mutation. Atherosclerosis.

[8] Virmani R, Burke AP, Farb A, Kolodgie FD (2006). Pathology of the vulnerable plaque. J Am Coll Cardiol.

[9] Falk E, Shah PK, Fuster V (1995). Coronary plaque disruption. Circulation.

